# Determination of spectral resolutions for multispectral detection of apple bruises using visible/near-infrared hyperspectral reflectance imaging

**DOI:** 10.3389/fpls.2022.963591

**Published:** 2022-08-29

**Authors:** Insuck Baek, Changyeun Mo, Charles Eggleton, S. Andrew Gadsden, Byoung-Kwan Cho, Jianwei Qin, Diane E. Chan, Moon S. Kim

**Affiliations:** ^1^Environmental Microbial and Food Safety Laboratory, Agricultural Research Service, U.S. Department of Agriculture, Beltsville, MD, United States; ^2^Department of Biosystems Engineering, College of Agriculture and Life Sciences, Kangwon National University, Chuncheon, South Korea; ^3^Interdisciplinary Program in Smart Agriculture, Kangwon National University, Chuncheon, South Korea; ^4^Department of Mechanical Engineering, University of Maryland-Baltimore County, Baltimore, MD, United States; ^5^Department of Mechanical Engineering, McMaster University, Hamilton, ON, Canada; ^6^Department of Biosystems Machinery Engineering, College of Agricultural and Life Science, Chungnam National University, Daejeon, South Korea

**Keywords:** apple, bruise, support vector machine, discriminant analysis, hyperspectral image

## Abstract

This study demonstrates a method to select wavelength-specific spectral resolutions to optimize a line-scan hyperspectral imaging method for its intended use, which in this case was visible/near-infrared imaging-based multiple-waveband detection of apple bruises. Many earlier studies have explored important aspects of developing apple bruise detection systems, such as key wavelengths and image processing algorithms. Despite the endeavors of many, development of a real-time bruise detection system is not yet a simple task. To overcome these problems, this study investigated selection of optimal wavelength-specific spectral resolutions for detecting bruises on apples by using hyperspectral line-scan imaging with the Random Track function for non-contiguous partial readout, with two experimental parts. The first part identified key-wavelengths and the optimal number of key-wavelengths to use for detecting low-, medium-, and high-impact bruises on apples. These parameters were determined by principal component analysis (PCA) and sequential forward selection (SFS) with four classification methods. The second part determined the optimal spectral resolution for each of the key-wavelengths by selecting and evaluating 21 combinations of exposure time and key-wavelength bandwidths, and then selecting the best combination based on the bruise detection accuracies achieved by each classification method. Each of the four classification methods was found to have a different optimized resolution for high accuracy bruise detection, and the optimized resolutions also allowed for use of shorter exposure times. The results of this work can be used to help develop multispectral imaging systems that provide rapid, cost-effective post-harvest processing to identify bruised apples on commercial processing lines.

## Introduction

Bruises on apples have a significant impact on the quality of the fruit. In addition to introducing unpleasant sensory attributes and causing nutrient loss, bruises reduce customer preference and discourage repeat purchases, which can lead to substantial economic losses (Xing et al., 2007; ElMasry et al., 2008; Huang et al., 2015; Zhu et al., 2016). Hence, bruise detection is a crucial procedure during packaging and transport. Hyperspectral visible and near-infrared (VIS–NIR) imaging, which can simultaneously assess both chemical and physical properties of food materials, has played an increasingly important role in nondestructive classification technology over the past two decades and many published studies have reported on the application of VIS–NIR hyperspectral imaging for detecting bruises on apples. Six- and four-waveband combinations with principal component analysis (PCA) and/chemometric methods were suggested for detecting bruises on Jonagold and Golden Delicious apples (Xing et al., 2005, 2007; Xing and De Baerdemaeker, 2005). ElMasry et al. (2008) reported three NIR wavelengths to be effective for detecting bruises on McIntosh apples. Furthermore, the classification of apple bruising time using VIS–NIR hyperspectral image was also investigated (Zhang and Li, 2018; Pan et al., 2019). With the development of machine learning methods and improved computer hardware, more and more studies have been conducted using elaborate imaging algorithms for bruise detection. Che et al. (2018) reported a method for pixel-based extraction of apple bruise regions coupled with random forest, Tan et al. (2018) categorized four degrees of bruising with 92.5% accuracy by using support vector machine (SVM) based on grid search parameter optimization, and Zhu and Li (2019) detected and visualized slight bruises on apples with 92.9% accuracy using classification by extreme learning machine method (ELM). Keresztes et al. (2016, 2017) applied partial least-squares discriminant analysis (PLS-DA) with shortwave infrared hyperspectral imaging to overcome glare problems during apple image processing to detect bruises.

Most of these studies ultimately aimed to build rapid screening systems. However, even though the fundamental requirements have been studied, such as identifying key wavelengths, developing image processing algorithms, and selecting light sources, the application and development phase to implement rapid screening using multispectral imaging is not trivial. Actual application of the previous knowledge to implement real-time rapid screening systems requires additional research. Thus, this study specifically aims to determine optimal bandwidths of spectral images used for a given specific application to improve image acquisition time and accuracy of classification algorithms, and specifically for optimizing multispectral real-time implementation—critical to effective imaging-based apple bruise detection but also relevant to other applications.

The main challenge faced by many researchers stems from the pixel-readout rates of the conventional charge-coupled device (CCD) sensor, which are not fast enough for use in real-time-based systems (Yoon et al., 2011). Although hyperspectral VIS–NIR line-scan cameras predominantly utilize silicon-based CCD sensors for providing high-quality spectral images, electron-multiplying CCD (EMCCD) sensors have been developed to overcome the problem regarding read-out rate. The EMCCD capacity for non-contiguous partial readout implies that only a few specific wavelength lines in a frame could be chosen by the user for a fast frame rate as well as for reduction of the image data volume, rather than reading out the entirety of data across all wavelength lines detected. Therefore, when using EMCCD sensors, this function, called random-track (RT) mode, is essential to developing a rapid multispectral line-scan system for use on commercial processing lines (Kim et al., 2003, 2005, 2007; Baek et al., 2019). In addition to enabling hyperspectral operation as a multispectral imaging device, RT mode can choose the region of interest (ROI) in the spectral direction for each waveband selected. Thus, the ROIs in the spectral direction have the effect of a binning method, which provides several advantages for developing a rapid screening system. The main benefit is reducing the data size per line-scan image in ordinary computer memory, leading to a greater number of lines scanned per second (Greensill and Walsh, 2000; Lu, 2003). Moreover, the effects of binning include smoothing of the spectra as a spectral pretreatment and correcting of small peak shifts, both of which help prevent spurious correlations with signal noise (Kim et al., 2008, 2011; Anderson et al., 2011).

For applying RT mode, the size of the ROI in the spectral direction should be optimized since it influences the spectral resolution of the waveband images and the dynamic data range of the analog-to-digital (A/D) converter. These two factors are crucial when analyzing spectrum data and making a detection model as well as when developing a rapid sorting system. The wide dynamic data range provides a more detailed description of the target in the image, and using appropriate spectral resolution can prevent information overload and drive a fast response by allowing more light to reach the image sensor effectively. So far, there has been little discussion about the spectral resolution and dynamic data range of the A/D digitizer as dependent on the size of the ROI in the spectral direction (Greensill and Walsh, 2000; Kim et al., 2011), although the use of filters (Kodak Wratten 82C gelatin filters) to attenuate the CCD response level at specific wavelengths for acquiring quasi-optimized wavelength-dependent system throughput and for using a greater portion of the 14-bit dynamic range of the A/D digitizer at multiple wavelengths has been reported (Kim et al., 2011).

The primary aim of this study is to explore the optimized spectrum resolution in the spectral direction by RT mode of the VIS–NIR hyperspectral camera for detecting bruises on apples. To be specific, this study focus on: (1) the selection of key-waveband images for detecting apple bruises by using PCA and sequential forward selection (SFS); (2) the optimization of the size of the ROI in the spectral direction based on key-waveband images; and (3) the investigation of the ROI size in the spectral direction with SVM and discriminant analysis for creating an efficient model and a rapid multispectral line-scan imaging system for the real-time processing operation. Thereby, this paper systematically shows that multi-band line-scan imaging systems can be optimized for their intended application.

## Materials and methods

### Preparation of apples

“Golden Delicious” apples were purchased from a local market in two separate batches, each in advance of one of the two planned experiments (key-waveband selection and optimization of spectral resolution) needed to create the apple bruise detection model. For the first experiment, 120 apples manually selected for their lack of any apparent surface defects were carefully packed into trays to prevent bruising during transportation from market to laboratory. The apples were divided into four groups of 30 each, for use in three levels of physical impact testing along with one control group of unbruised apples (high, medium, low, and sound), and each apple was labeled for identification. For the second experiment, a total of 24 apples was purchased and divided into four groups of six apples each, again for high-, medium-, and low-impact bruising groups and one unbruised control group.

### Bruising procedure

**Figure 1:**
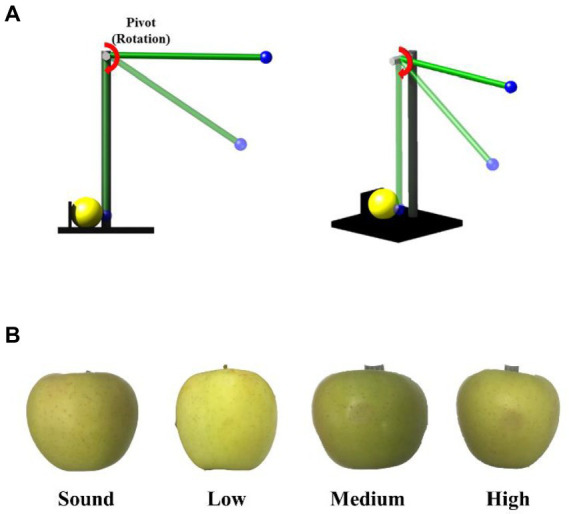
**(A)** Schematic of the apple-bruising device and **(B)** color image of sound and bruised samples.

Figure 1 illustrates the equipment for developing bruises on apples. A 67 g steel ball was mounted onto the steel rod that was 68 cm in length and 663 g in weight. The other end of the steel rod was mounted onto a pivot. Thus, the ball and rod act as a simple pendulum that can be used to create apple bruises by physical impact. Such devices have been used for generating bruises in apples in previous studies (Xing et al., 2006; Opara et al., 2007). After an apple was placed in a sample holder, the pendulum was released, accelerated under the influence of gravity, and impacted at the apple’s equator. Three different levels (1.11, 0.66 and 0.33 J) of impact energy were utilized to induce high-, medium-, and low-impact bruises. The corresponding three initial angles for the pendulum were 50, 38, and 27 degrees, respectively. The impact energy E (in J) was calculated by Equation 1 (Zhu et al., 2016). After subjecting each apple to one impact, the impact area on the apple surface was marked using a sticker for subsequent reference when evaluating detection results, and the apples were stored at room temperature (22°C) for the 24-h testing period during which the apples were imaged multiple times.


(1) $$E=m_{b}gh+m_{r}g\frac{h}{2}$$

Where *m*_b_ and *m*_r_ are the masses of the steel ball and the steel rod (kg), respectively; *g* is the gravity of acceleration (m/s^2^), and *h* is the height (m) of the steel ball from ground.

### Hyperspectral imaging system

**Figure 2:**
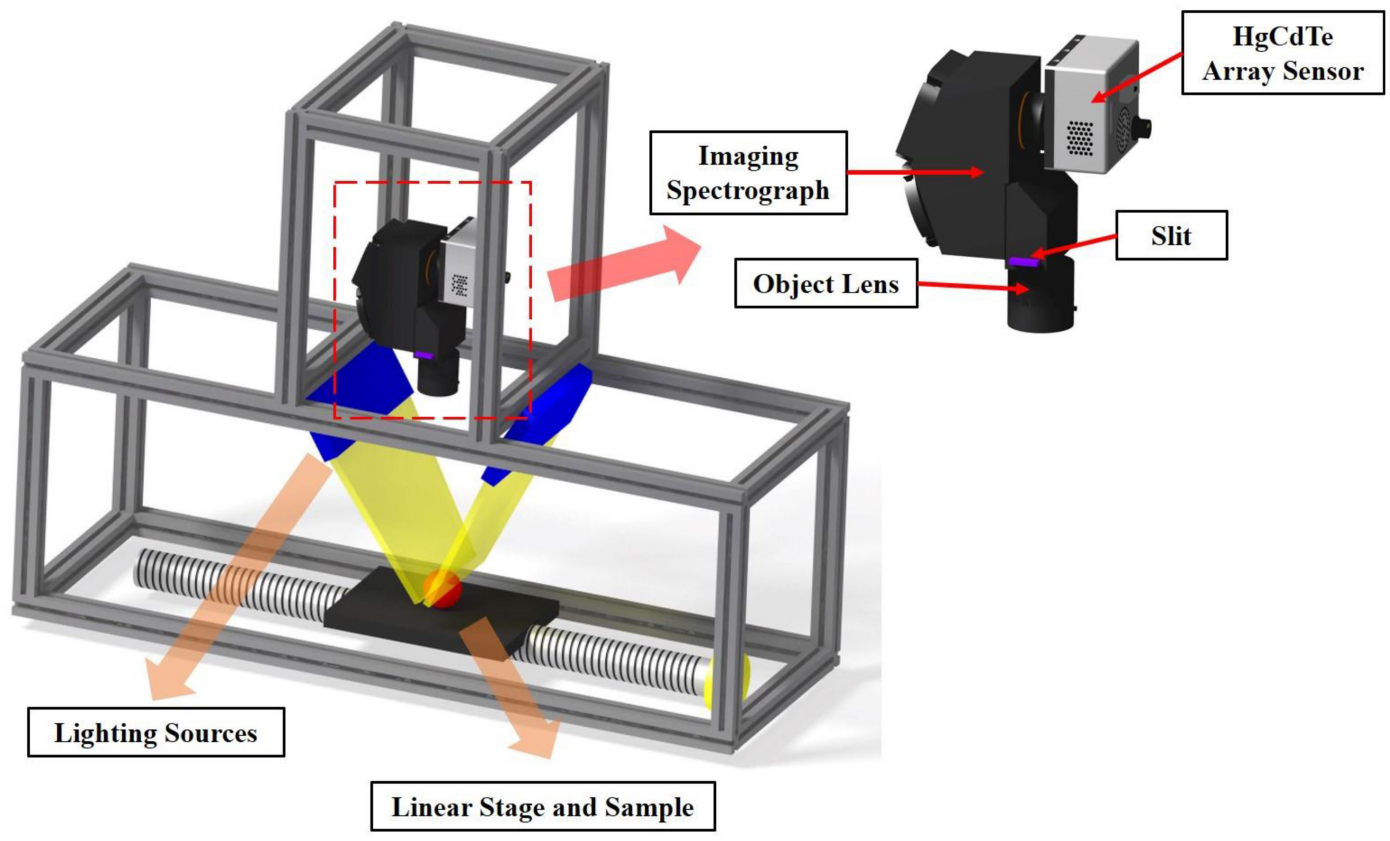
Schematic of hyperspectral imaging system and the hyperspectral camera.

Hyperspectral images of the apples were acquired by using the line-scan (pushbroom) hyperspectral imaging system illustrated in Figure 2. The system consisted of an electron-multiplying charge-coupled device (EMCCD) camera (EMCCD: Luca R DL-604M, 14-bit, Andor Technology, South Windsor, CT, United States), visible/near-infrared imaging spectrograph (Headwall photonics, Fitchburg, MA, United States), a programmable linear stage (translation table) with stepping motor, and light sources. The camera was coupled with a C-mount objective lens (F1.9 35-mm compact lens, Schneider Optics, Hauppauge, NY, United States). The hyperspectral imaging system was constructed to cover visible to near-infrared wavelengths for reflectance measurements. The lighting sources used were two 150-W halogen lamps with DC power supplies which enabled control of light intensity. Light was transmitted *via* two optical fibers to provide near-uniform illumination. Hyperspectral reflectance images of apple samples were collected by placing the loaded apple sample plate onto the programable translation table unit and obtaining spectral/spatial data line-by-line as the translation table moved the sample plate under the instantaneous field of view (IFOV) of the hyperspectral imaging system.

To extract the actual spectral response of the samples, the VIS–NIR hyperspectral images were calibrated by a flat-field method. When using a hyperspectral imaging camera, the raw hyperspectral images contain noise and artifacts related to the measurement environment and imperfections of each component (e.g., source, lens, filter, spectrograph, and camera). By using flat-field correction, noise was reduced and the relative reflectance spectrum data from hyperspectral image was gained (Kim et al., 2001). To be specific, the relative reflectance intensity (I_R_) can be determined by the following process (Kim et al., 2001). White and dark reference images were acquired after collecting hyperspectral data for individual sample plates. A white reference was obtained using a Spectralon panel (~99% reflectance, SRT-99-120, Labsphere, North Sutton, NH, United States), and the dark reference was obtained by capping the objective lens.


(2) $$I_{R}=\frac{I_{r}-I_{d}}{I_{w}-I_{d}}$$

### Selection of key-wavelengths

**Figure 3:**
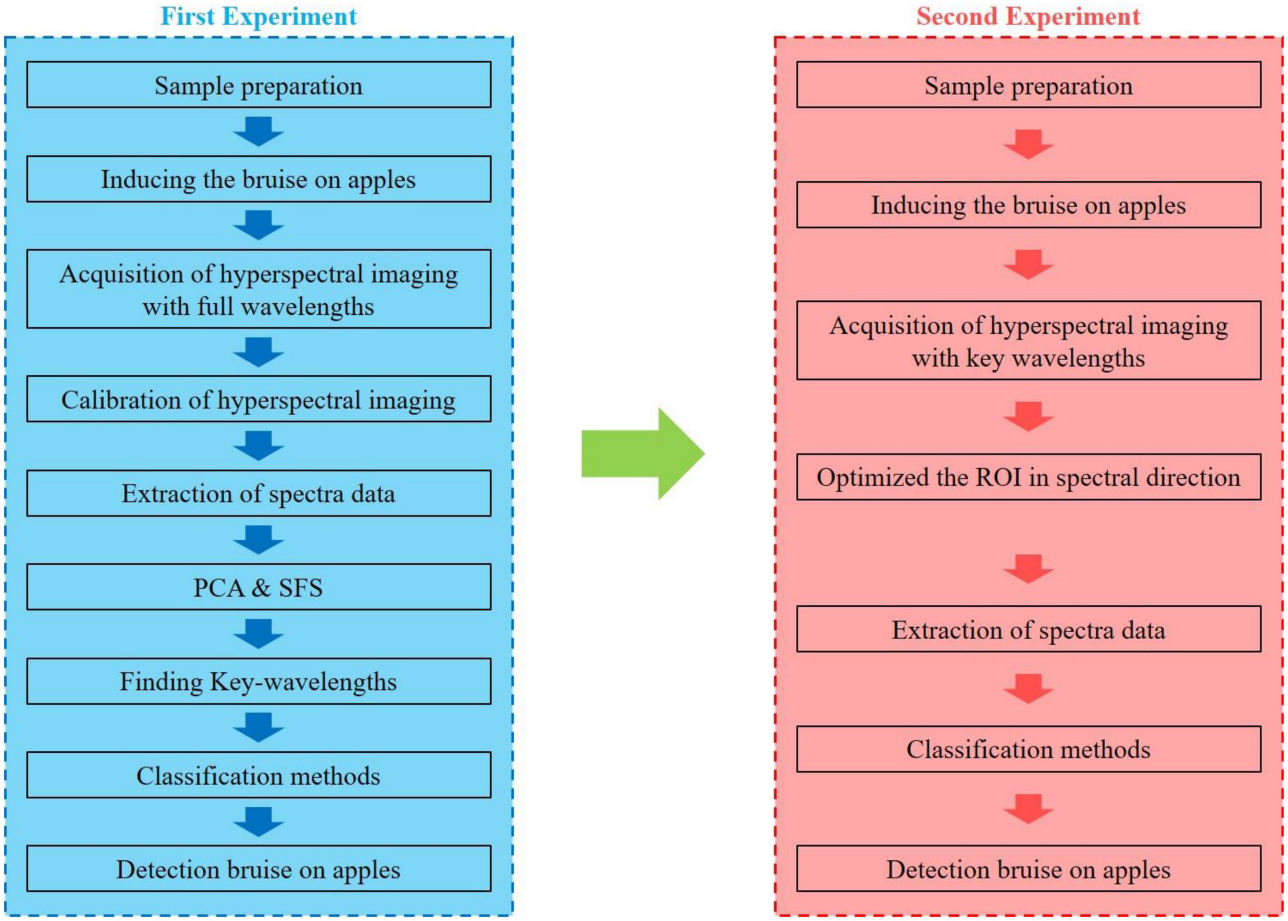
Key steps in the procedure used to find optimal wavelengths and spectrum resolutions to detect bruises on apples.

The steps of the procedure for finding key wavelengths and optimal wavelength resolutions for detecting apple bruises are shown in Figure 3. This study had two experimental parts. The first experiment was conducted to find key-wavelength to detecting bruise and second experiment was conducted to search for optimum wavelength resolution based on key wavelength. In the first experiment, hyperspectral images of apples were obtained and spectral data for bruised and sound regions of the apples were extracted from the images. Because the shape of the bruise on each apple was different and the apple bruises were not consistently classifiable by any one single wavelength image, manual pixel selection was used to visually identify pixels from clearly observable bruised and sound regions in the third or fourth principal component (PC3 or PC4) images, such as those shown in Supplementary Figure 1, that were created based on entire wavelength images.

Spectral data were then extracted from the raw hyperspectral images at those bruised and sound pixels identified from the PC 3 or 4 images. Next, the extracted spectral data were split into a training set (70%) and a test set (30%) to be used for modeling and generalization, respectively. The optimum number of wavelengths and the key wavelengths for detecting bruises on apples were selected by SFS with four classifiers. Before applying SFS, a pre-selection of wavelengths was chosen by PCA weighting coefficients because the training for the model would take a long time if the entire spectral range of the hyperspectral wavelength images were to be used. The PC images were created by linear transformation. Therefore, the peaks and valleys for each weighting coefficient of the PCs imply a dominant wavelength, which means that the dominant wavelengths have the largest contribution when performing the linear transformation (Cho et al., 2013).

Thus, predominant wavelengths were first pre-selected by PCA, and then from this pool, optimal key wavelengths and the optimum number of key wavelengths to use were identified by using SFS with classifiers. After identifying key-wavelengths, SVM and a discriminant analysis model was optimized based on key-wavelengths and optimum number of wavelengths. When making classification models, all of the methods were coupled with a five cross-validation schemes to prevent overfitting problems. In this study, two results were shown based on spectrum data and hyperspectral images. The models were applied to both spectrum data and hyperspectral images in generating the results. Details of the principles and equations of each analysis method are explained in the Supplementary material.

### Optimized parameters for hyperspectral imaging

As mentioned before, the CCD responses in the spectral and spatial domains are not uniform. For the purpose of quasi-optimizing the wavelength-dependent system throughput and gaining a greater portion of the dynamic range of the A/D digitizer, the system throughput can be compensated by optimizing the ROI in the spectral direction (spectrum resolution). For example, the multispectral imaging system can use three specific wavebands selected for optimal target detection. These three wavebands may each have different quantum efficiency (QE). The exposure time can be set to use the full A/D dynamic data range for the first waveband which has the highest QE. However, the remaining second and third wavebands (each with lower QE than the first waveband) will not optimally use their full A/D dynamic data range since these two wavebands may have lower response for the same exposure time. If exposure time were to be set to maximize dynamic data range at the second or third wavebands, then the signal at the first waveband would be overexposed (saturated signal). Therefore, the procedure to optimize spectrum resolution must be done carefully since spectrum resolution is associated with target detection accuracy and image acquisition time in conjunction with exposure time. Thus, crucial to the process of choosing the size of the ROI in the spectral direction is the trade-off between each key wavelength resolution and image acquisition time.

To find optimum spectrum resolutions and exposure time, the second experiment in this study used two sets of combinations based on key-wavelength spectrum resolution. The first and second sets for wavelengths were based on four classifiers, respectively. The CCD responsivity was highest at 553.9 nm among the key-wavelengths. Exposure time was selected by maximizing the dynamic data range centered at 553.9 nm. Therefore, the wider the bandwidth at 553.9 nm, the shorter the exposure time. The bandwidths of each of the remaining two key-wavelengths were selected by dynamic data range. For example, with a narrow bandwidth at 553.9 nm, an exposure time of 0.17 s was needed to use the full dynamic range. However, the remaining two wavebands could not reach full dynamic range in only 0.17 s. So, the bandwidths for the remaining two wavebands were adjusted to reach 25, 50, or 90% of their dynamic data ranges, in 21 combinations of the three wavebands. Tables 1, 2 show the 21 parameter combinations used to optimize the bandwidths. After using the 21 imaging parameter combinations to take hyperspectral images of the bruised and sound apples according to the steps described in Figure 3, a model was made for each parameter combination.

**Table 1:** Combinations of wavelength resolutions and exposure times for QDA and SVM-RBF classifier.

Combination number	Exposure time (s)	Centered 774.2 nm		Centered 553.9 nm		Centered 424.5 nm	
		Size of the ROI	Dynamic range (%)	Size of the ROI	Dynamic range (%)	Size of the ROI	Dynamic range (%)
1	0.17	0	90%	0	90%	7.8	90%
2	0.012	11.7	90%	10.2	90%	73.5	90%
3	0.012	7.0	50%	10.2	90%	46.9	50%
4	0.012	0.8	25%	10.2	90%	10.2	25%
5	0.0048	27.4	90%	25.0	90%	112.6	90%
6	0.0048	14.9	50%	25.0	90%	73.5	50%
7	0.0048	3.9	25%	25.0	90%	14.9	25%
8	0.002	58.6	90%	50.0	90%	162.6	90%
9	0.002	35.2	50%	50.0	90%	104.7	50%
10	0.002	7.0	25%	50.0	90%	22.7	25%

**Table 2:** Combinations of wavelength resolutions and exposure times for LDA and SVM classifier.

Combination number	Exposure time (s)	Centered 812.5 nm		Centered 553.9 nm		Centered 424.5 nm	
		Size of the ROI	Dynamic range (%)	Size of the ROI	Dynamic range (%)	Size of the ROI	Dynamic range (%)
11	0.17	0.8	90%	0.0	90%	7.8	90%
12	0.17	0.0	50%	0.0	90%	4.7	50%
13	0.012	18.8	90%	10.2	90%	73.5	90%
14	0.012	12.5	50%	10.2	90%	46.9	50%
15	0.012	1.6	25%	10.2	90%	10.2	25%
16	0.0048	41.4	90%	25.0	90%	112.6	90%
17	0.0048	25.8	50%	25.0	90%	73.5	50%
18	0.0048	47	25%	25.0	90%	14.9	25%
19	0.002	76.6	90%	50.0	90%	162.6	90%
20	0.002	44.6	50%	50.0	90%	104.7	50%
21	0.002	13.3	25%	50.0	90%	22.7	25%

### Image processing

One of the advantages of hyperspectral imaging is that it provides a visualization map for the samples being imaged. Using the combined characteristics from the acquired spatial and spectral information, the developed four classification models which are linear discriminant analysis (LDA), quadratic discriminant analysis (QDA), SVM, and SVM with radial basis function kernel (RBF-SVM), can be applied to hyperspectral images to form classification maps. A simple classification based on the intensity of the pixels can be obtained. In this study, the visualization process was performed on hyperspectral images of apples surfaces with the background removed by applying the different classification models. The resultant images provide information that can be used to determine the presence of any bruises on an apple surface image. Since a classification model will assign lower score values to pixels in bruise regions and higher score values to pixels in sound regions, the resultant images can be used for discriminating between bruised and sound region on apples.

## Results and discussion

### Average spectra of bruise on apple

**Figure 4:**
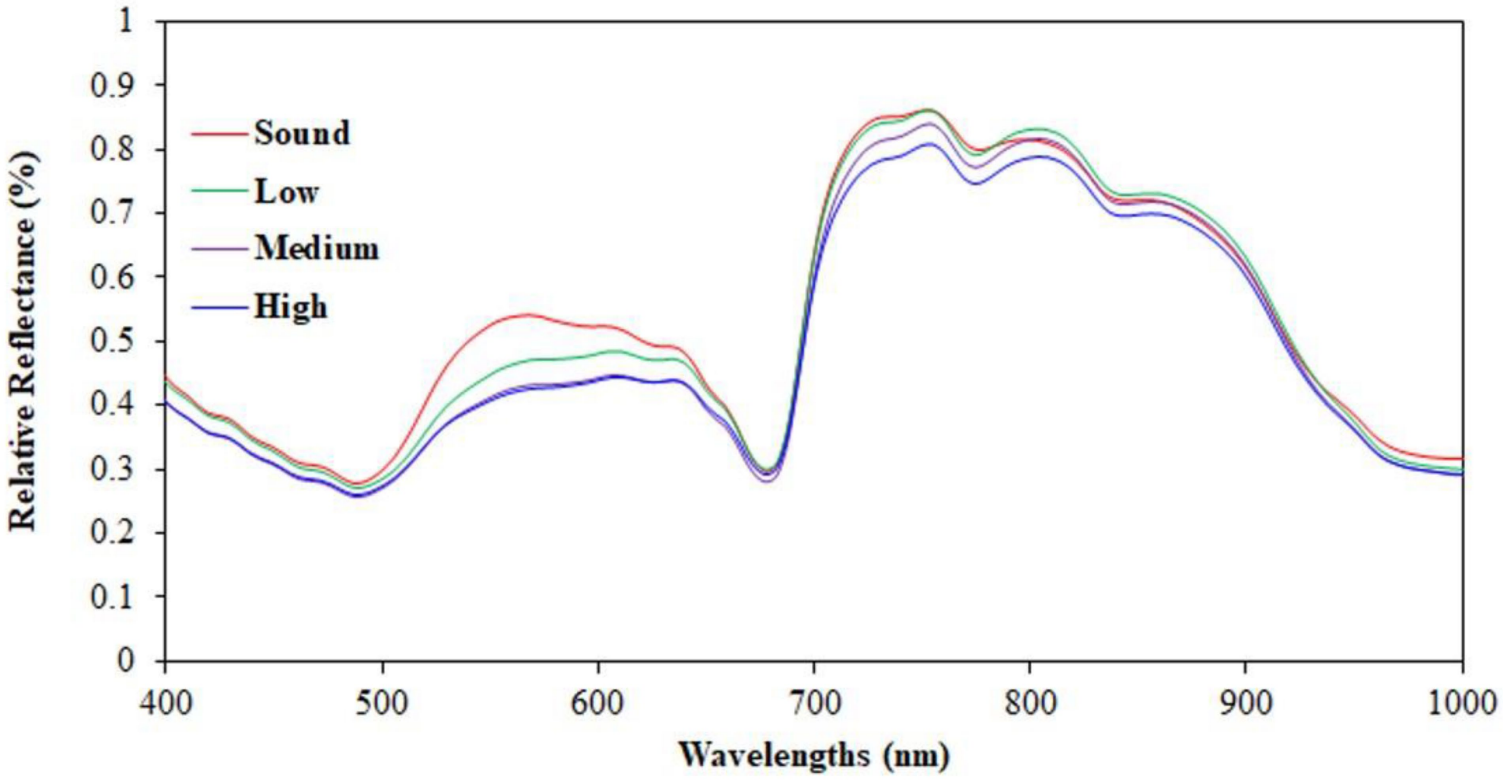
Averaged relative reflectance of sound apple regions and of bruised regions caused at low-, medium-, and high-impact energy levels.

Figure 4 presents the average spectra of bruised and sound regions, in different colors, from the hyperspectral images acquired. In general, a wavelength around 558 nm indicated the browning symptom of the bruised region on an apple (Xing et al., 2005). The color of bruised apple areas was browner and darker compared to sound areas, and the corresponding differences in reflectance intensity between sound areas and impacted areas is clearly seen in the wavelengths between 550 and 600 nm in Figure 4. Differences in reflectance were observed for the three different impact energy levels. The reflectance of high-impact bruises was lower than the reflectance of medium-and low-impact bruises since the color and chemical compound change on an apple surface occurred the most quickly from bruising at the high-impact energy level. Absorption valleys around 500 (450–550 nm) and 680 nm were exhibited, which relate to the carotenoids and chlorophyll pigment in apple peel, respectively (Baranowski et al., 2012). The sugar content of apples was reflected in the absorption valley at 820 nm (Zhang and Li, 2018). When an apple bruise develops, the cells in the apple tissue are damaged and the intercellular air spaces decrease, causing differences in water content between bruised and sound regions (ElMasry et al., 2008). Hence, the relative reflectance of bruised regions is lower than in sound regions at wavelengths associated with water absorption, since the bruised regions have increased water content. The NIR region from 750 to 1,000 nm, which is associated with the water absorption wavebands (ElMasry et al., 2008), presented predominant wavebands for classifying bruises. In addition to identifying water absorption, these wavebands are also free of color information, and thus would be useful for detecting differences between bruised and sound regions regardless of apple color (Huang et al., 2015). The relative reflectance spectra in Figure 4 illustrate spectral indicators of carotenoids and chlorophyll pigment in apple peel (450–550 nm), sugar content (820 nm), and water absorption (750–1,000 nm) associated with the phenomenon of bruises on apple. Therefore, bands selected in these spectral regions would be useful for a multispectral imaging system to detect bruises.

### Key-wavelength selection

If the entire spectrum of wavelengths were to be used for choosing the key-wavelengths, training a model with the amount of data obtained would take a long time. Therefore, this study used PCA to pre-select a pool of potential key-wavelengths. In this study, the first through fourth principal components were used since PC1 through PC4 accounted for 99.21% of the spectrum data variation as shown in the Supplementary Figure 2. Moreover, visual evaluation by PC image suggested that images beyond PC4 contained no useful data variance for detecting bruised regions. Supplementary Figure 1 shows PC images for one apple that was subjected to high-impact bruising. Characteristically, the PC1 images showed explanation for the largest variance of the data, which were influenced largely by the surface variance of the apples. The PC2 images display some dark spots that resulted from the absorption of chlorophyll. The PC3 images appear to show the greatest contrast between bruised and sound regions of the apple. Thus, PC3 images have high discrimination power for classifying the bruised regions from sound regions and would contain key wavelengths. The PC4 images show some evidence of the presence of bruise regions, but both sound and bruise regions have a darker appearance with less contrast, which would be less useful for distinguishing between bruise and sound regions.

**Figure 5:**
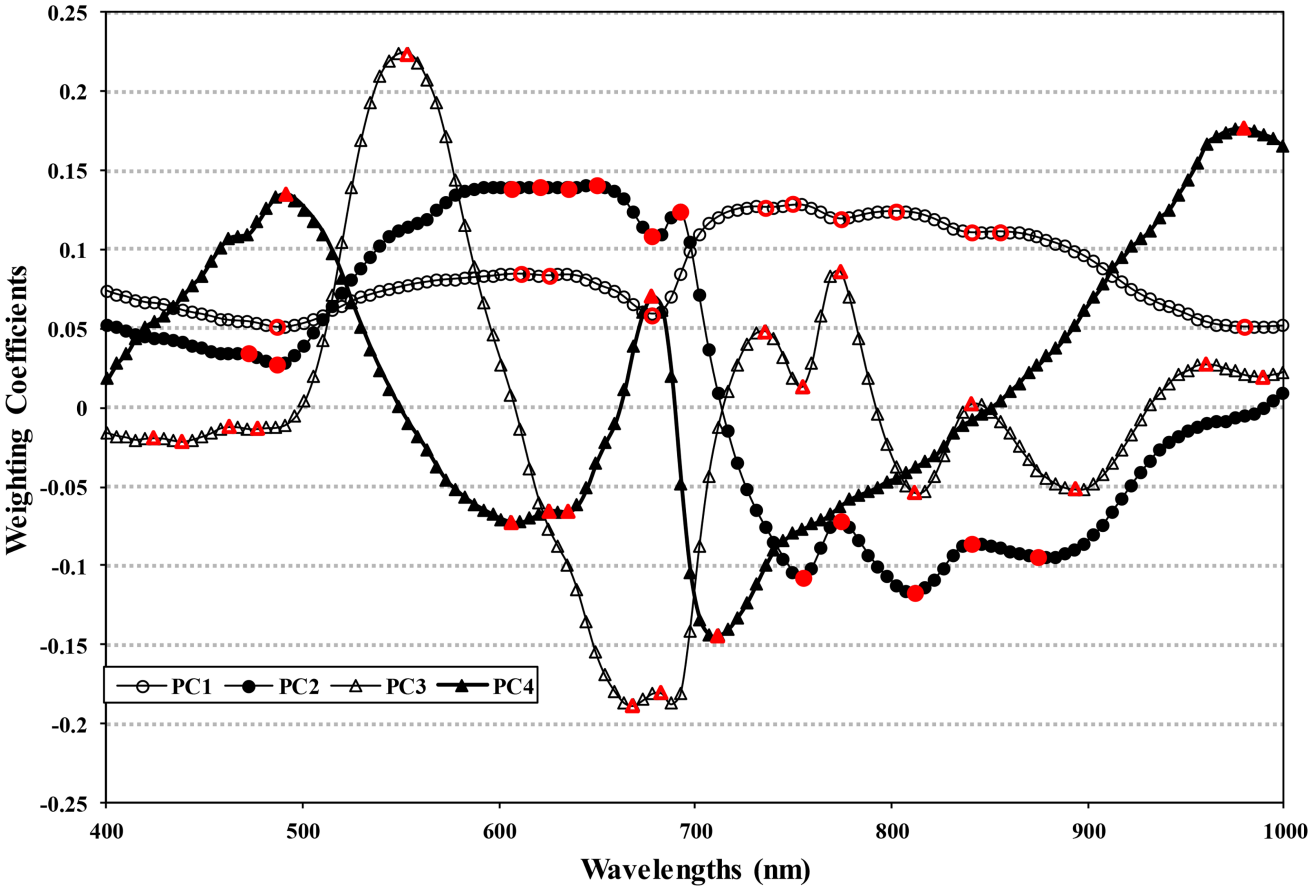
Spectral weighting coefficients for the first, second, third, and fourth principal components, with dominant wavebands for pre-selection marked in red.

Figure 5 shows the weighting coefficients for PC1 through PC4, obtained from the entire range of the reflectance hyperspectral images. The red markers in Figure 5 indicate the dominant wavelengths of each PC. Supplementary Table 1 lists the dominant wavelengths for each PC and the potential key-wavelengths that were pre-selected based on the weighting coefficients. Similar to results observed for the average spectrum data, the pre-selected wavelengths included wavelengths associated with known absorptions for carotenoids, chlorophyll, sugar, and water. Bruising has known effects on these components in apple tissue. These results suggest that the wavelengths pre-selected through PCA are suitable as potential key-wavelength selections.

**Figure 6:**
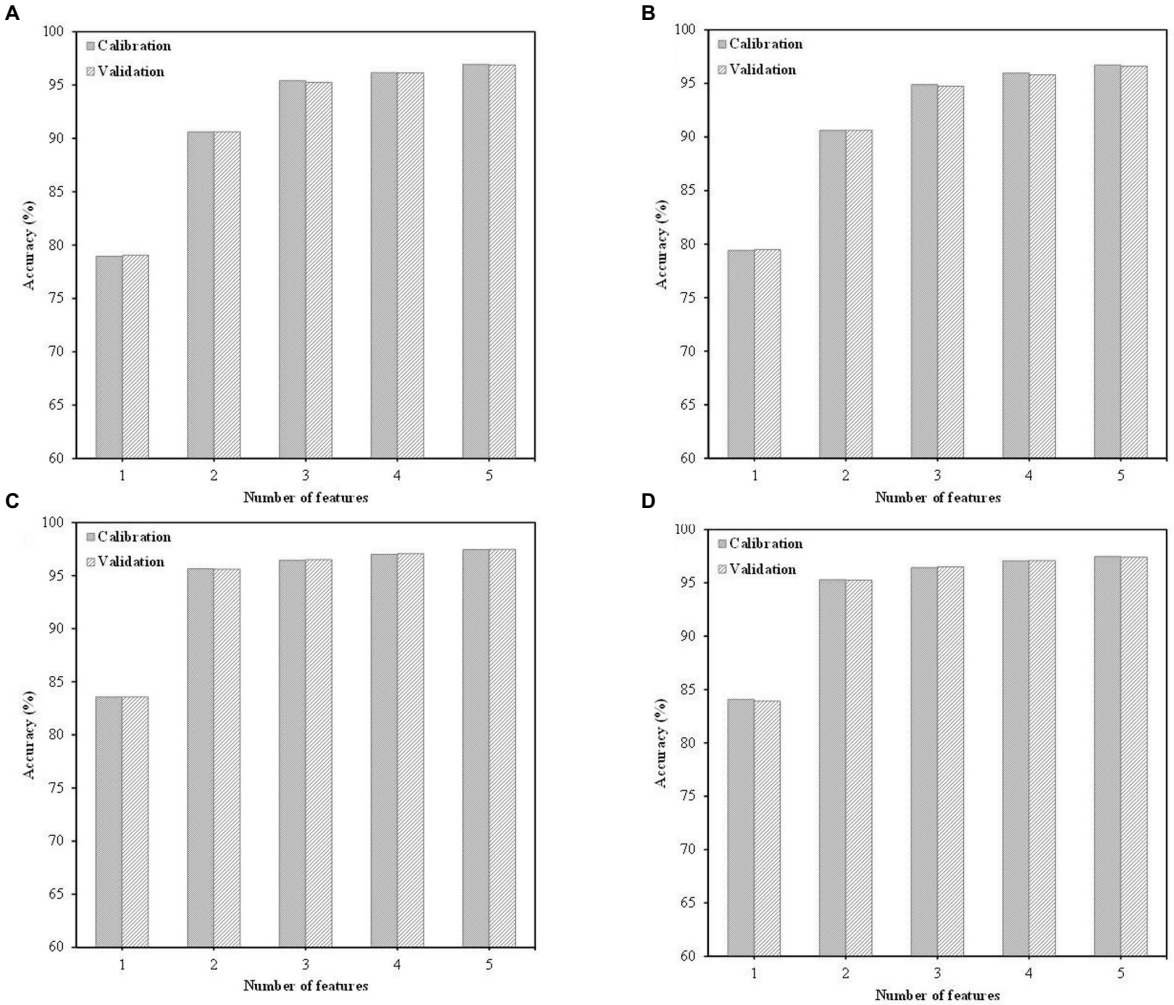
Performance comparison of sequential forward selection (SFS) using four classifiers: **(A)** support vector machine (SVM) with slack variable; **(B)** linear discriminant analysis (LDA); **(C)** SVM with radial basis function (RBF) kernel; and **(D)** quadratic discriminant analysis (QDA).

The optimum number of wavelengths for detecting bruises on apples was determined by the SFS method based on the PCA pre-selected wavelengths. Figure 6 presents the bruise detection accuracies corresponding to the number of wavelengths used, from 1 to 5, for the four classifiers. The SVM and LDA classifiers showed lower accuracies (near 90%) when using only two features, compared to the SVM-RBF and QDA classifiers which demonstrated 95% accuracy with only two features used. With three or more features used, all of the classifiers obtained higher accuracies (over 94%). Therefore, in this study, the number of optimal wavelengths was considered to be three wavelengths for further analysis of wavelength resolutions, since there was no significant improvement in classification accuracy when more wavelengths were added beyond three. The results of identifying the key-wavelengths for classifying bruised regions on apples are shown in Table 3, and are similar to the previous result from PC image analysis. The PC3 image has higher discriminant power than other PCs image with fewer components. The weighting coefficients of PC3 included 424, 553, 774, and 812 nm as the predominant wavelengths. Furthermore, these wavelengths for detecting bruises were also observed by Solovchenko et al. (2010). According to their observations, brownish pigment exhibits increasing absorption monotonously from NIR to shorter wavelengths, and they suggested three wavelengths around 550, 750, and 800 nm as key wavelengths. The 550 nm wavelength was sensitive to browning symptoms and the 750 nm wavelength exhibits less variation in sound regions than bruised regions. Another spectral feature characteristic of the key-wavelengths is the association of flavonols and anthocyanin with wavelengths near 420 and 810 nm, respectively.

**Table 3:** The three most important wavelengths for each classifier, as determined by the sequential forward selection (SFS) method for selection of key-wavelengths.

Classifier	First wavelength (nm)	Second wavelength (nm)	Third wavelength (nm)
SVM	812.5	553.9	424.5
SVM with RBF	553.9	424.5	774.2
LDA	812.5	553.9	424.5
QDA	553.9	424.5	774.2

### Optimum resolution for detecting bruises on apples

**Figure 7:**
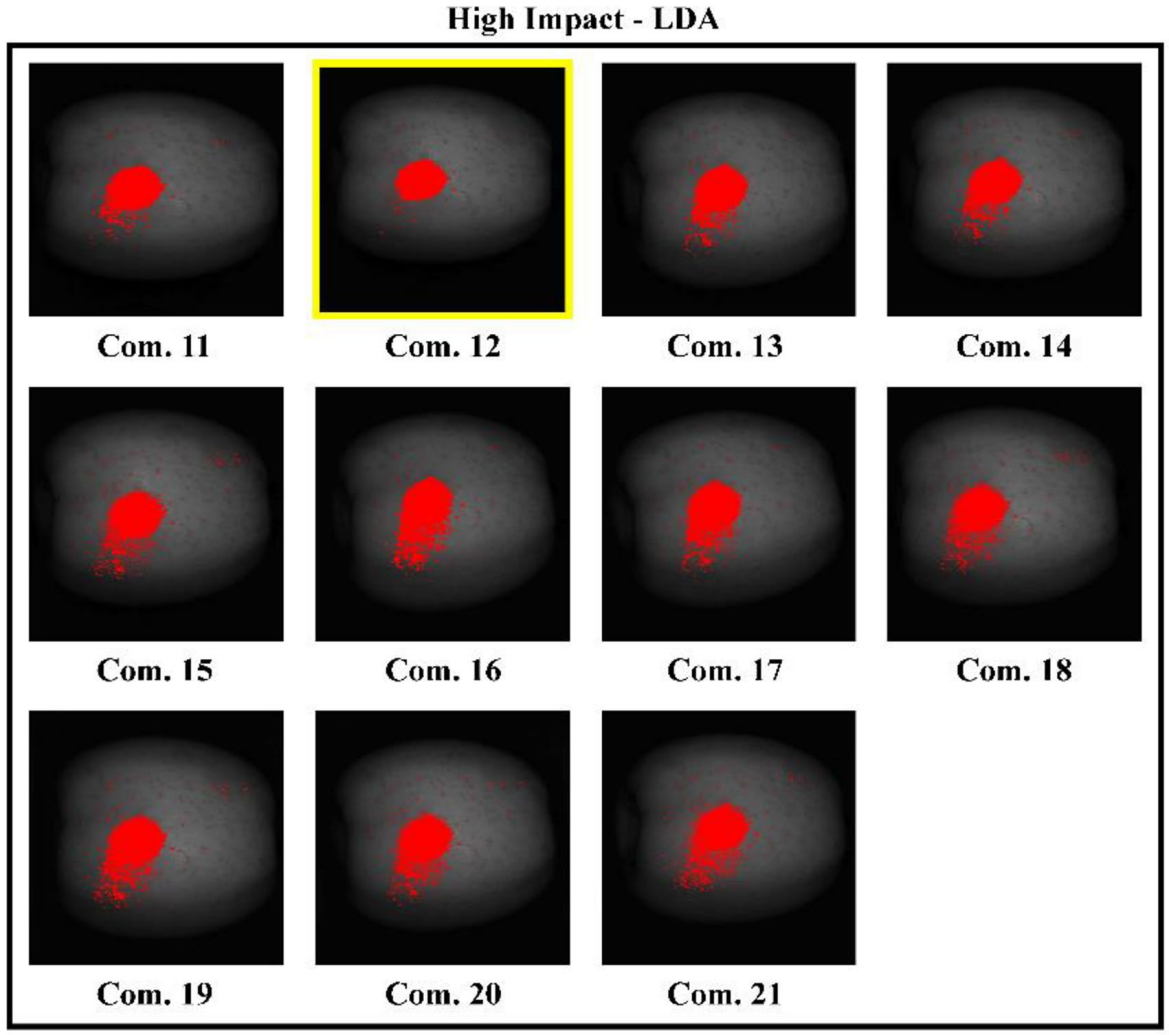
LDA classification result images using various wavelength resolutions, for apples with high-impact level bruises. Combination 12 (boxed in yellow) was found to be the best wavelength resolution for detecting bruises.

After obtaining multispectral images of the apples using the 21 bandwidth combinations listed in Tables 1, 2, the classification result images were generated by applying the models for visualizing the bruises on apples. For LDA, combination #12 (0.12 s exposure time; 812.5, 424.5, and 553.9 nm throughputs at 50, 50, and 90% of dynamic range, respectively) showed the best result image, with the fewest number of scattered pixels of false positives. Figure 7 shows the LDA classification result images for apples with high-impact bruises. All of these images show false positives but combination #12 has the smallest misclassification area. All QDA classification result images exhibited good visual classification. The QDA combination #9 image (0.002 s exposure time; 774.2, 424.5, and 553.9 nm throughputs at 50, 50, and 90% dynamic range, respectively) produced relatively good classification results compared with LDA combination #12. QDA combination #9 detects the bruise well and the scattered pixels of false positives around the bruise region are the fewest. Moreover, exposure time is shorter for QDA combination #9 than for LDA combination #12, indicating that QDA combination #9 can be used to take more images than the LDA method in any given time period. The SVM resultant images are similar to the LDA resultant images. The SVM classification results are the best for combination #12. This result implies that linear type classification methods are influenced by 553.9 and 424.5 nm as these wavebands represents the brown symptom of bruise and are identically employed by both models. In contrast with the QDA result, the optimum parameters for SVM-RBF result images were in combination #6 (0.0048 s exposure time; 774.2, 424.5, and 553.9 nm throughputs at 50, 50, and 90% of dynamic range, respectively). Other combination result images show many scattered pixels of false positives around bruise region. The discriminant power of SVM-RBF is higher at combination #6. In addition to bandwidth, dynamic range also had noticeable effect. The QDA and SVM-RBF models showed their best results at 50% of dynamic range at other two key-wavelength; at over 50%, misclassification of pixels increased. If dynamic ranges are over 50%, then misclassification of pixels increases.

### Classification result with optimized resolution

**Figure 8:**
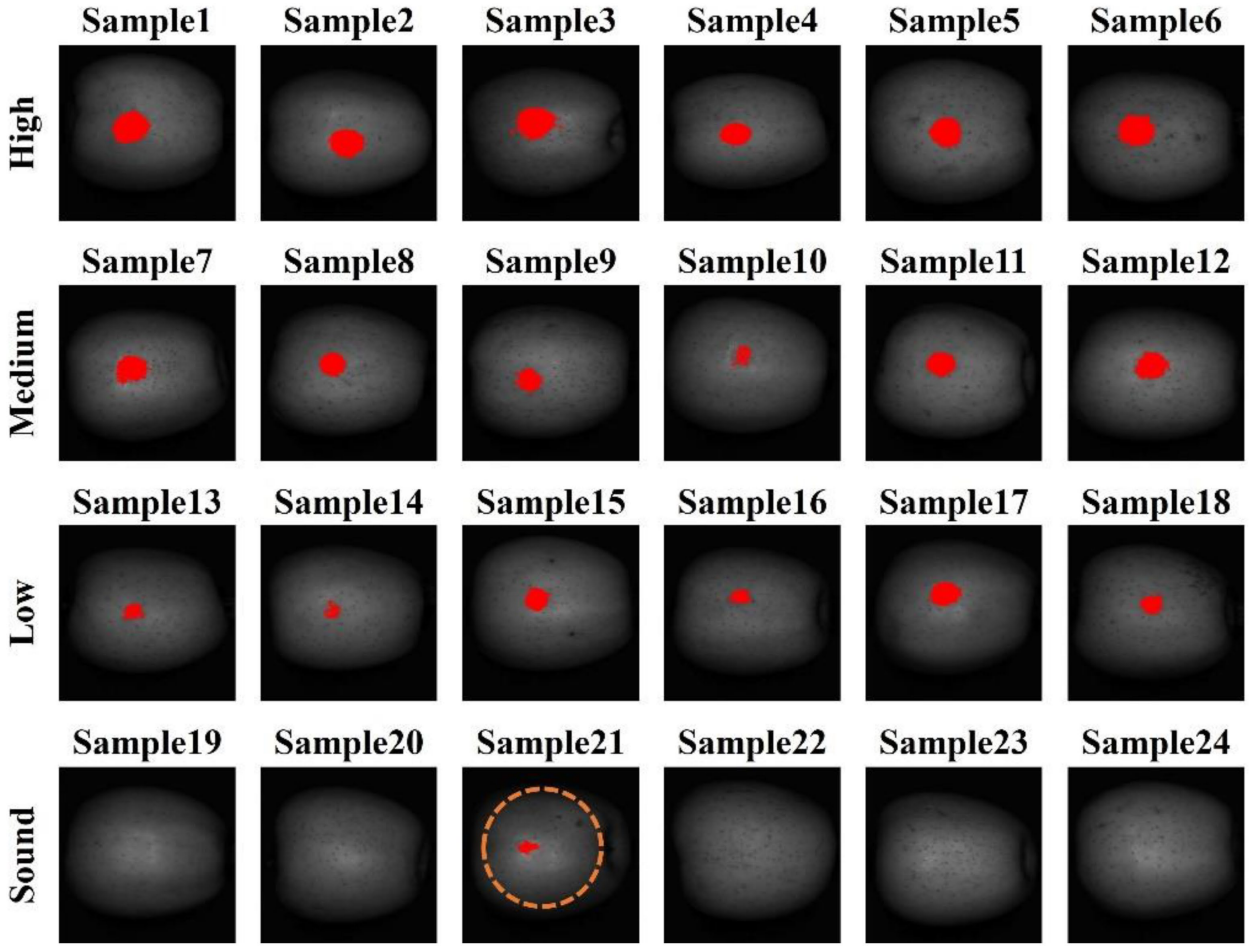
Bruise classification images from LDA model with combination #12.

Resultant images for all 24 apples are shown in Figure 8 for the LDA classification model using combination #12, which yielded the best LDA-based results. This model led to a false positive on sample 21, but correctly detected the bruised and sound areas on the other 23 apples. The background regions were removed from the 553.9 nm waveband image by using a masking method, and then the classification models were applied to three band images. When applying classification methods, the model was only applied to apple area pixels, not the entire image, to lower image processing time. After applying the classification method, only those pixels identified as bruise region pixels have a value of “1” in the binary image. Some scattered pixels of false positives in the classification images were eliminated by open-close image processing methods, and then this image was used as reference image for further processing to visualize the bruise against the sound surface of the apple. Based on reference image, the bruised region pixels are changed to red color to reveal them in the 553.9 nm band image, and most were successfully detected on the apple surface as shown in Figure 8. Upon application of the algorithm to the multispectral images of the 24 apples, more than 90% of the bruised apples were correctly identified. Therefore, using a broad bandwidth is efficient since broad bandwidth can reduce acquisition time and maximize dynamic range, and thus is suitable for rapid line-scan-based sorting systems for real-time processing. For classification of bruised and sound apple surfaces, parameter combinations # 6, #9, and #12 were found to result in the highest accuracies overall. However, the QDA model with combination #9 yielded better (faster) performance speed in detecting bruises, compared to the other classification methods with their best combinations. The bandwidths of the key-wavelengths investigated in this study could be helpful for developing rapid multispectral devices for monitoring apple quality. The classification resultant images for QDA, SVM, and SVM-RF with optimized spectrum resolutions are shown in Supplementary Figures 3–5, respectively.

## Conclusion

In this research, a large multispectral image data set was constructed for Golden Delicious apples subjected to low-, medium-, and high-impact bruising, key-wavelengths for bruise detection were selected, and wavelength-specific spectral imaging resolution was evaluated based on classification results for multiple combinations of exposure time and spectral bandwidths. Four classification methods were applied to multispectral images spanning a range of resolutions, and the best resolution and dynamic range were selected in terms of the ability to identify bruises on apples. The most significant and important result of this study is finding optimum bandwidths and dynamic range. The LDA and SVM, have their best performances at parameter combination #12. These combination parameters can use over 90% of the full-dynamic range. The QDA and SVM-RBF are suitable for detecting bruises on apples using parameter combinations #9 and #6, respectively. This part of the research evaluated diverse combination parameters related to acquisition speed and the wavelength-dependent system throughput of camera CCD sensors in conjunction with the use of four classification algorithm. The results showed that the QDA classification method with parameter combination #9 could achieve 90% accuracy with the shortest exposure time (0.002 s). The method developed here for determining appropriate bandwidths can be applied to many more industry applications beyond detection of apple bruises and will be of interest to researchers and developers faced with the task of reducing system speeds.

## Data availability statement

The original contributions presented in the study are included in the article/Supplementary material; further inquiries can be directed to the corresponding authors.

## Author contributions

IB: conceptualization, methodology, formal analysis, investigation, data curation, visualization, writing—original draft, and writing—review and editing. CM: conceptualization, formal analysis, writing—review and editing, supervision, and funding acquisition. CE: methodology, validation, writing—original draft, and writing—review and editing. SG: methodology, writing—original draft, and writing—review and editing. B-KC: methodology, validation, data curation, and visualization. JQ: validation, investigation, and data curation. DC: methodology and writing—review and editing. MK: conceptualization, writing—review and editing, supervision, project administration, and funding acquisition. All authors contributed to the article and approved the submitted version.

## Funding

This work was carried out with the support of “Cooperative Research Program for Agriculture Science and Technology Development (Project No. PJ01709504)” from the Rural Development Administration, South Korea.

## Conflict of interest

The authors declare that the research was conducted in the absence of any commercial or financial relationships that could be construed as a potential conflict of interest.

## Publisher’s note

All claims expressed in this article are solely those of the authors and do not necessarily represent those of their affiliated organizations, or those of the publisher, the editors and the reviewers. Any product that may be evaluated in this article, or claim that may be made by its manufacturer, is not guaranteed or endorsed by the publisher.
