# Supplementary material for: Determination of spectral resolutions for multispectral detection of apple bruises using visible/near-infrared hyperspectral reflectance imaging
Source: Front Plant Sci. 2022 Aug 29;13:963591. doi: 10.3389/fpls.2022.963591 (PMC9465011; doi:10.3389/fpls.2022.963591)
Supplement: Supplementary file 1 [file Data_Sheet_1.docx]

#### **Principal component analysis (PCA)**

Principal Component Analysis (PCA) is a typical feature extraction method. PCA was used for the entire spatial-spectral dimension data of each apple. Each PC component was made through a linear transformation. The transformation vector (W) was calculated based on a covariance matrix of the spectrum data. The detailed process is explained below in several steps.

First Step: the mean value $(u_{x})$ and covariance $\left( \Sigma_{x} \right)$ of X feature matrix are calculated.

$$u_{x}=\frac{1}{N}\sum_{i=1}^{N} x_{i}$$

$$\Sigma_{x}=\frac{1}{N}{\left( X-M_{x} \right)\left( X-M_{x} \right)}^{T}, M_{x}=u_{x}1^{T}$$

Where 1 is an n dimension column vector such that every component of the vector has a value of one.

Second Step: Eigenvectors$(U)$ of $\Sigma_{x}$, and diagonal matrix $(\Lambda)$ of $\Sigma_{x}$ whose diagonal elements are the eigenvalues corresponding to each eigenvector, are calculated.

$$\Sigma_{x}=U\Lambda U^{T}=\left[ u_{1},u_{2},\cdots u_{n} \right]\left[ \begin{matrix} \lambda_{1} & 0 & \ldots& 0 \\ 0 & \lambda_{2} & \ldots& 0 \\ \vdots& \vdots& \ldots& \vdots\\ 0 & 0 & \ldots& \lambda_{n} \end{matrix} \right]\left[ u_{1},u_{2},\cdots u_{n} \right]^{T}$$

Third Step: m number of eigenvalues ($\left\{ \lambda_{1},\lambda_{2},\lambda_{3},\cdots,\lambda_{m} \right\}$) are selected in order from the largest to smallest eigenvalue.

Fourth Step: a transformation matrix (W) that is a column vector is generated by (equivalent to) the eigenvector which corresponds to the selected eigenvalue.

$$w=\left[ u_{1}{,u}_{2},\cdots,u_{m} \right]$$

Fifth Step: new feature matrix (Y) is obtain by multiplying the transform of W with X.

$$Y=W^{T}X$$

**Support vector machine (SVM)**

The ultimate aim of this study was to identify the optimal key-wavelengths for a classification model that will discriminate between sound and bruised surfaces of an apple. Therefore, a support vector machine (SVM) multivariate analysis-based classification model was considered in this study. SVM has been widely used in many areas including optical character recognition and object recognition, since it is a promising non-parametric and non-linear classification technique (Auria & Moro, 2009; Baumann et al., 2018; Saitta, 1995). The SVM finds the best hyperplane, known as decision boundary, in feature dimensional space. The best hyperplane has good separation and has the largest margin between groups, as the largest margin has a lower generalization error (Sanz et al., 2016). The SVM with a slack variable identifies the linear decision boundary depending on the value of C (slack variable). This decision boundary has a feature dimension that is one less than the original data. For example, if the original data has two dimensions, the decision boundary has a line (one dimension) called the decision boundary or hyperplane. In contrast to SVM with a slack variable, the non-linear SVM with a kernel function identifies a hyperplane in a higher dimensional feature space by using mapping methods. This paper used a Radial basis function (RBF) kernel which was expanded by equation 3 since it is very flexible and build up fast (Auria & Moro, 2009; Ropodi, Panagou, & Nychas, 2018).

$K\left( x_{i},x_{j} \right)=e^{{-(x_{j}-x_{i})}^{T}\gamma^{-2}\sum^{-1} (x_{j}-x_{i})/2}$ (3)

where Σ is the variance-covariance matrix of all training sets and γ is only parameter which is chosen by user. The kernel size of RBF is controlled by the value of γ. All of the parameters related to SVM methods are optimized through grid search methods.

**Discriminant analysis**

Linear discriminant analysis (LDA) and quadratic discriminant analysis (QDA) methods were considered as classifiers. LDA and QDA are simple and intuitive. LDA develops a model that minimizes the within-group variance while maximizing the between-group variance (Caporaso, Whitworth, Grebby, & Fisk, 2018; Sanz et al., 2016; Wakholi et al., 2018). QDA is close to LDA, except a covariance matrix must be estimated for each group. In this case, a decision boundary between groups is a quadratic surface. However, if the training data set does not follow a Gaussian distribution, then LDA and QDA will lead to erroneous results since these methods are based on the concept of Bayes’ theorem (Wakholi et al., 2018). Because of the effectiveness of these two techniques, numerous studies have used these methods in many fields. In agricultural fields, these methods have been used in pattern recognition and classification problems.

**Sequence forward selection (SFS)**

In this study, SFS with classifiers was applied to the calibration set to select the optimal variables for building a discriminative model to classify sound and bruised regions on apples. The approach starts with an empty set. During step one, all the variables that have not yet been selected are considered for selection, and their impact on the evaluation score are recorded. At the end of this step, the variables resulting in the best score are included in the set. Then a new step is started, and the remaining variables are considered. This process is repeated until a prespecified number of variables has been included in the set (Cen, Lu, Zhu, & Mendoza, 2016; Ortaç, Bilgi, Taşdemir, & Kalkan, 2016; Vélez Rivera et al., 2014). In this study, the hyperspectral imaging data has one hundred features. A long time would be required to conduct classification training, since SFS methods should consider all possible combinations with cross-validation method. SFS method was applied to pre-selected key wavelengths only, in order to reduce the workload and complexity.


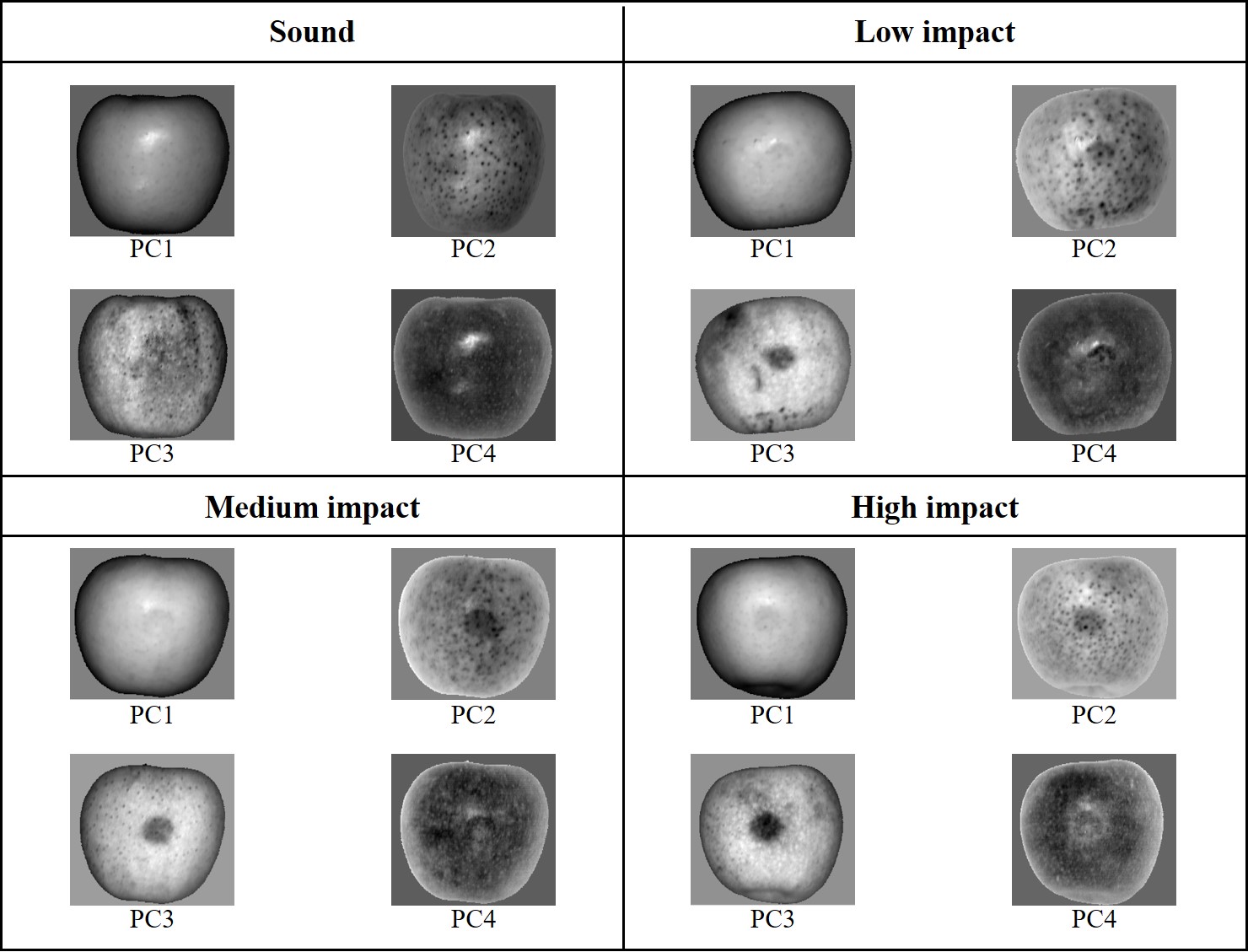


Supplementary figure 1. First through fourth PC images gained by using the entire spectral range from 400 to 1000 nm of the hyperspectral image data. The displayed PC images are for one sound (unbruised) apple and three apples subjected to low-, medium-, or high-impact bruising.


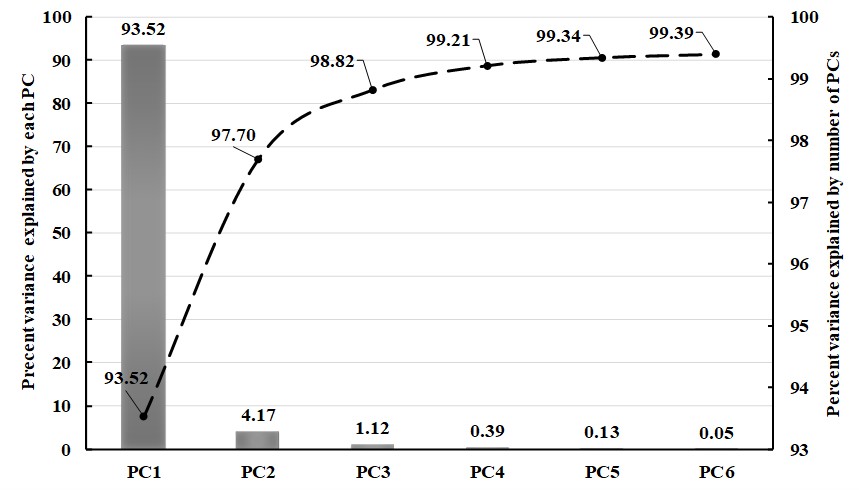


Supplementary figure 2. Column chart showing the share of each component in explaining the data variance (left y-axis), and line plot indicating the portion of the total variance (right y-axis) that is explained by *n* PCs.

Supplementary table 1. Dominant wavelengths for each of the first four PCs as determined by the valleys and peaks in the weighting coefficients.

|  | Dominant wavelengths (nm) |
| --- | --- |
| PC1 | 486.8, 611.3, 625.7, 678.4, 735.9, 750.2, 774.2, 802.9, 841.2, 855.6, 980.1 |
| PC2 | 472.4, 486.8, 606.5, 620.9, 635.3, 649.6, 678.4, 692.8, 755.0, 774.2, 812.5, 841.2, 874.8 |
| PC3 | 424.5, 438.9, 462.8, 477.2, 553.9, 668.8, 683.2, 735.9, 755.0, 774.2, 812.5, 841.2, 893.9, 961.0, 989.7 |
| PC4 | 491.6, 606.5, 625.7, 635.3, 678.4, 711.9, 980.1 |
| Pre-selected  wavelengths | 424.5, 438.9, 462.8, 472.4, 477.2, 486.8, 491.6, 553.9, 606.5, 611.3, 620.9, 625.7, 635.3, 649.6, 668.8, 678.4, 683.2, 692.8, 711.9, 735.9, 750.2, 755.0, 774.2, 802.9, 812.5, 841.2, 855.6, 874.8, 893.9, 961.0, 980.1, 989.7 |


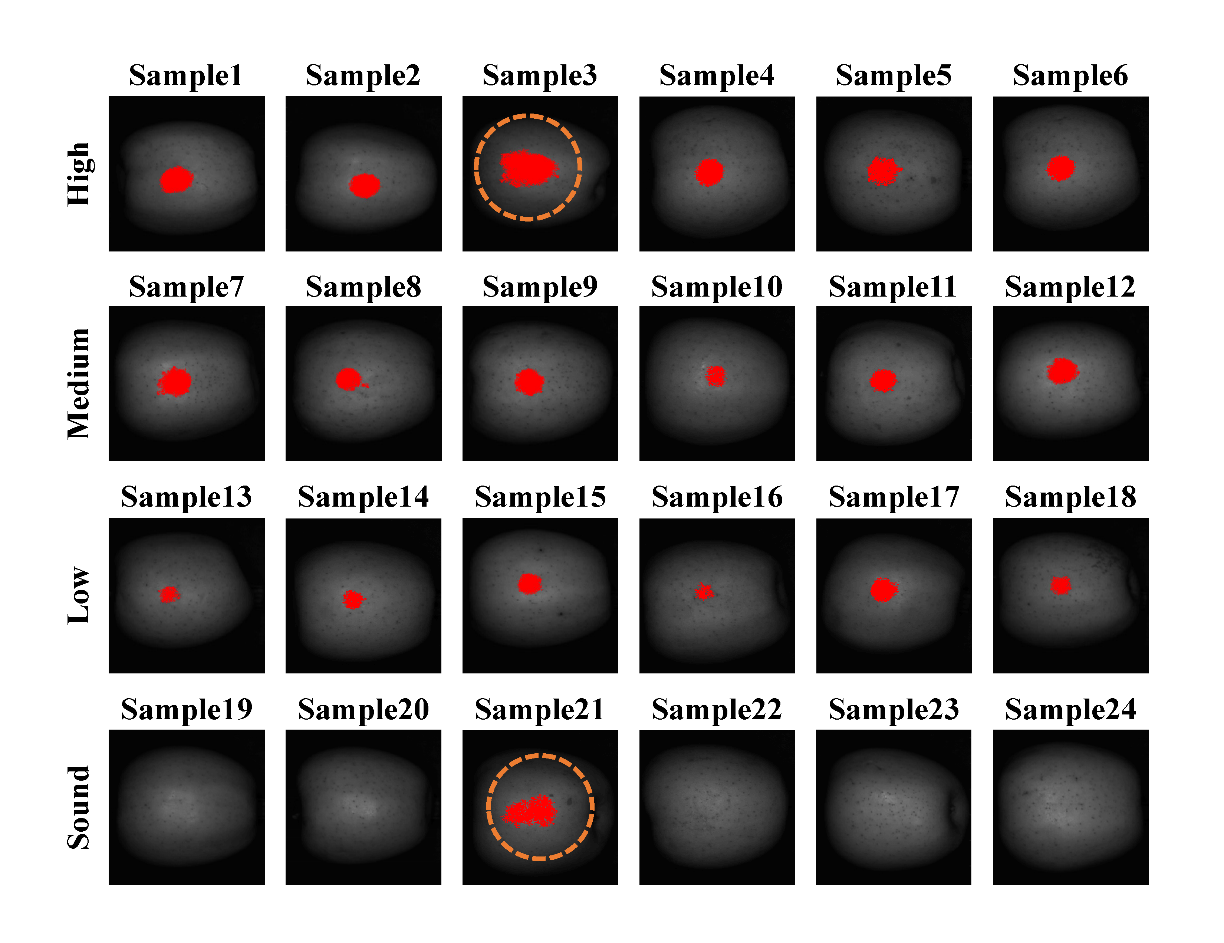


Supplementary figure 3. Classification resultant images for QDA with combination #9.


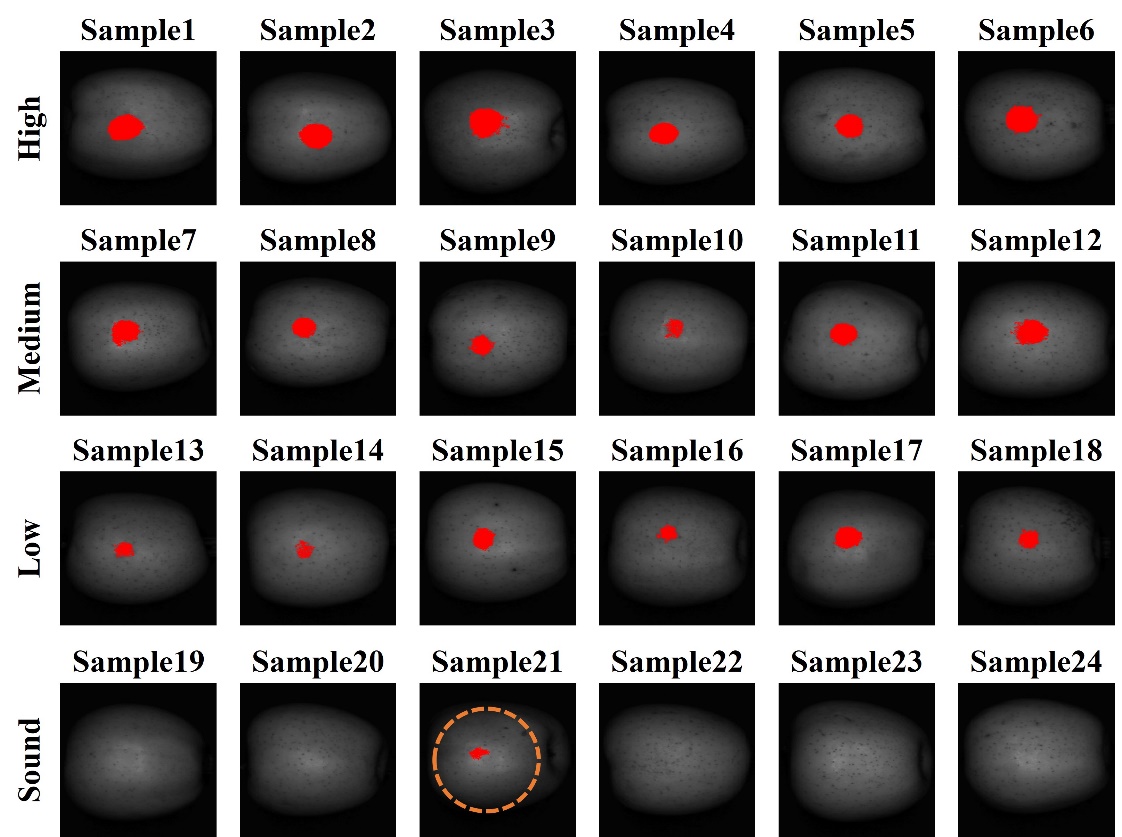


Supplementary figure 4. Classification resultant images for SVM with combination #12.


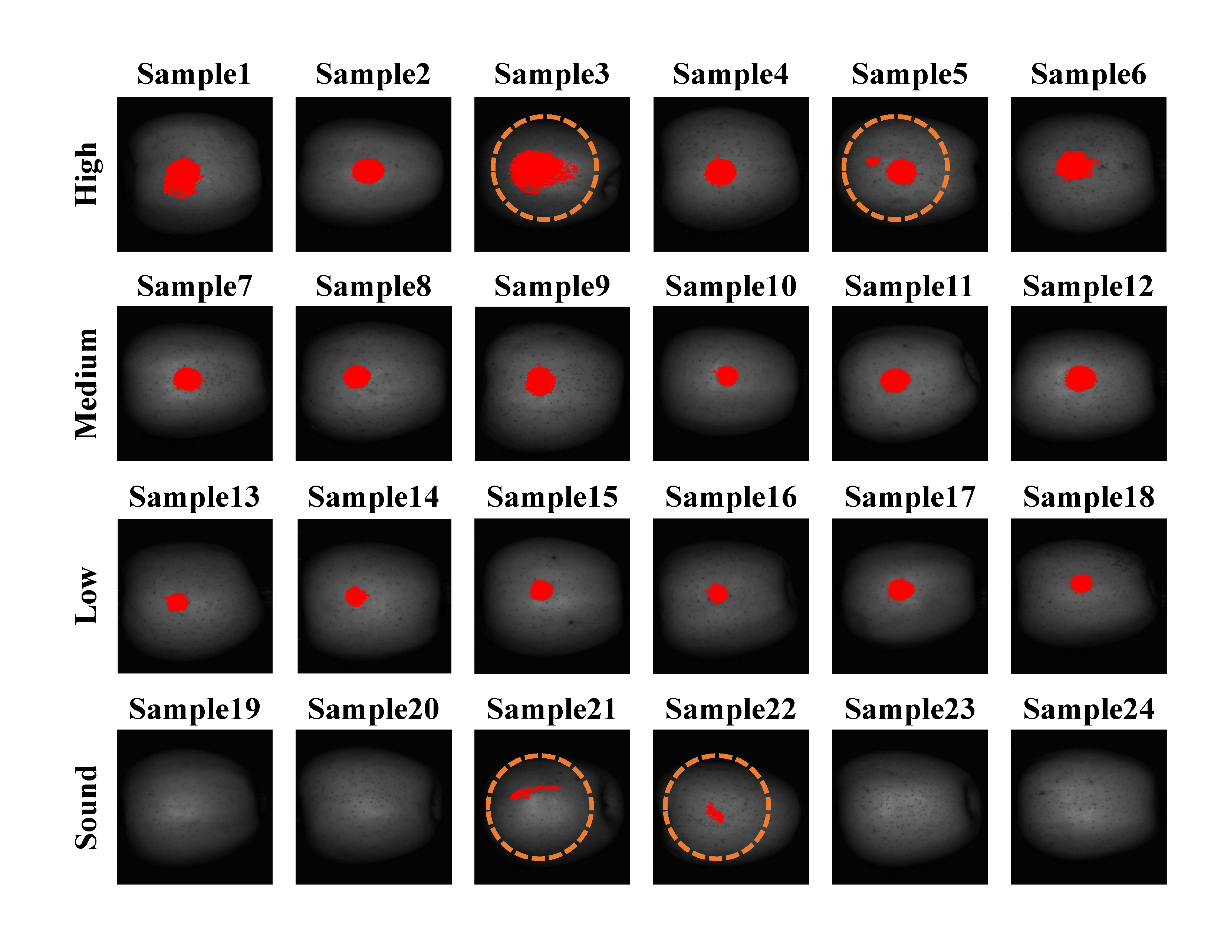


Supplementary figure 5. Classification resultant images for SVM-RBF with combination #6.

**References**

Auria, L., & Moro, R. A. (2009). Support Vector Machines (SVM) as a Technique for Solvency Analysis. *Ssrn*, (August). https://doi.org/10.2139/ssrn.1424949

Baumann, S., Groß, S., Voigt, L., Ullrich, A., Weymar, F., Schwaneberg, T., … Ulbricht, S. (2018). Pitfalls in accelerometer-based measurement of physical activity: The presence of reactivity in an adult population. *Scandinavian Journal of Medicine & Science in Sports*, *28*(3), 1056–1063. https://doi.org/10.1111/sms.12977

Caporaso, N., Whitworth, M. B., Grebby, S., & Fisk, I. D. (2018). Rapid prediction of single green coffee bean moisture and lipid content by hyperspectral imaging. *Journal of Food Engineering*, *227*, 18–29. https://doi.org/10.1016/j.jfoodeng.2018.01.009

Cen, H., Lu, R., Zhu, Q., & Mendoza, F. (2016). Nondestructive detection of chilling injury in cucumber fruit using hyperspectral imaging with feature selection and supervised classification. *Postharvest Biology and Technology*, *111*, 352–361. https://doi.org/10.1016/j.postharvbio.2015.09.027

Ortaç, G., Bilgi, A. S., Taşdemir, K., & Kalkan, H. (2016). A hyperspectral imaging based control system for quality assessment of dried figs. *Computers and Electronics in Agriculture*, *130*(3), 38–47. https://doi.org/10.1016/j.compag.2016.10.001

Ropodi, A. I., Panagou, E. Z., & Nychas, G. J. E. (2018). Rapid detection of frozen-then-thawed minced beef using multispectral imaging and Fourier transform infrared spectroscopy. *Meat Science*, *135*(September 2017), 142–147. https://doi.org/10.1016/j.meatsci.2017.09.016

Saitta, L. (1995). Support-Vector Networks SVM.pdf, *297*, 273–297. https://doi.org/10.1007/BF00994018

Sanz, J. A., Fernandes, A. M., Barrenechea, E., Silva, S., Santos, V., Gonçalves, N., … Melo-Pinto, P. (2016). Lamb muscle discrimination using hyperspectral imaging: Comparison of various machine learning algorithms. *Journal of Food Engineering*, *174*, 92–100. https://doi.org/10.1016/j.jfoodeng.2015.11.024

Vélez Rivera, N., Gómez-Sanchis, J., Chanona-Pérez, J., Carrasco, J. J., Millán-Giraldo, M., Lorente, D., … Blasco, J. (2014). Early detection of mechanical damage in mango using NIR hyperspectral images and machine learning. *Biosystems Engineering*, *122*, 91–98. https://doi.org/10.1016/j.biosystemseng.2014.03.009

Wakholi, C., Kandpal, L. M., Lee, H., Bae, H., Park, E., Kim, M. S., … Cho, B. K. (2018). Rapid assessment of corn seed viability using short wave infrared line-scan hyperspectral imaging and chemometrics. *Sensors and Actuators, B: Chemical*, *255*, 498–507. https://doi.org/10.1016/j.snb.2017.08.036
